# Early Detection of Sage (*Salvia officinalis* L.) Responses to Ozone Using Reflectance Spectroscopy

**DOI:** 10.3390/plants8090346

**Published:** 2019-09-12

**Authors:** Alessandra Marchica, Silvia Loré, Lorenzo Cotrozzi, Giacomo Lorenzini, Cristina Nali, Elisa Pellegrini, Damiano Remorini

**Affiliations:** 1 Department of Agriculture, Food and Environment, University of Pisa, Via del Borghetto 80, 56124 Pisa, Italy; alessandra.marchica@agr.unipi.it (A.M.); s.lore@studenti.unipi.it (S.L.); giacomo.lorenzini@unipi.it (G.L.); cristina.nali@unipi.it (C.N.); elisa.pellegrini@unipi.it (E.P.); damiano.remorini@unipi.it (D.R.); 2 CIRSEC, Centre for Climate Change Impact, University of Pisa, Via del Borghetto 80, 56124 Pisa, Italy; 3 Nutrafood Research Center, University of Pisa, Via del Borghetto 80, 56124 Pisa, Italy

**Keywords:** antioxidant capacity, aromatic plants, spectral phenotyping, non-enzymatic antioxidants, oxidative stress, partial least squares regression, partial least squares discriminant analysis, permutational multivariate analysis of variance, principal coordinates analysis, vegetation indices

## Abstract

Advancements in techniques to rapidly and non-destructively detect the impact of tropospheric ozone (O_3_) on crops are required. This study demonstrates the capability of full-range (350–2500 nm) reflectance spectroscopy to characterize responses of asymptomatic sage leaves under an acute O_3_ exposure (200 ppb for 5 h). Using partial least squares regression, spectral models were developed for the estimation of several traits related to photosynthesis, the oxidative pressure induced by O_3_, and the antioxidant mechanisms adopted by plants to cope with the pollutant. Physiological traits were well predicted by spectroscopic models (average model goodness-of-fit for validation (*R*^2^): 0.65–0.90), whereas lower prediction performances were found for biochemical traits (*R*^2^: 0.42–0.71). Furthermore, even in the absence of visible symptoms, comparing the full-range spectral profiles, it was possible to distinguish with accuracy plants exposed to charcoal-filtered air from those exposed to O_3_. An O_3_ effect on sage spectra was detectable from 1 to 5 h from the beginning of the exposure, but ozonated plants quickly recovered after the fumigation. This O_3_-tolerance was confirmed by trends of vegetation indices and leaf traits derived from spectra, further highlighting the capability of reflectance spectroscopy to early detect the responses of crops to O_3_.

## 1. Introduction

Tropospheric ozone (O_3_), produced by a variety of precursors, such as nitrogen oxides (NO_x_) and volatile organic compounds (VOCs), under light conditions, is a major phytotoxic air pollutant, with deleterious effects also on animal health [1,2,3]. Although numerous attempts have been made to attenuate the emission of its precursors, levels of O_3_ are still high in many areas of the world (e.g., North America, Europe, and South and East Asia) and are expected to increase further due to the presence of climatic changes and to anthropogenic activities [4], especially in hot-spot areas such as the Mediterranean basin [5]. However, decreases in surface O_3_ concentrations have been also reported in some Mediterranean sites [6]. The deleterious effects of excessive O_3_ uptake on plants include reduction of photosynthesis and growth, partial stomatal closure, cell dehydration, excessive excitation energy, accelerated leaf senescence, and appearance of chlorotic/necrotic leaf injuries, that overall result in reduction of crop production and huge economic loss [1]. Although O_3_ is commonly debated as a major air pollutant with detrimental effects on plants, some studies have also proposed exposure to an adequate concentration of O_3_ for a short time, under controlled conditions, as a tool to improve plant nutritional quality since it usually increases plant antioxidants to cope with O_3_-induced oxidative stress without severely affecting plant performance [7,8]. Thus, advancements in phenotyping techniques able to early detect and monitor the effects of O_3_ on crops, also in the absence of visible symptoms, may optimize crop management, potentially leading to reduced crop yield losses and increased crop quality.

Vegetation spectroscopy is an emerging sensor technology which, due to the advancements in the sensitivity and portability of instruments, as well as in progresses in computational capacity and chemometric modelling methods, has enabled the rapid and non-destructive estimation of several plant chemical and physiological traits based on the optical properties of living vegetation. The estimation of these traits from leaf reflectance depends on the exploitation of the relationships of light with molecular organic bonds, primarily C–H, N–H, and O–H, resulting in vibrational excitation at specific wavelengths of the electromagnetic spectrum, namely, the visible (VIS; 400–700 nm), the near-infrared (NIR; 700–1100 nm), and the short-wave infrared (SWIR; 1100–2400 nm) spectral regions [9,10].

The exploitation of reflectance spectra can be performed by simple vegetation indices based on the ratio of reflected light at different wavelengths that have been developed to predict foliar traits related, for example, to the structure of vegetation, the biochemistry and physiology of plants or their stress conditions (e.g., normalized difference vegetation index, NDVI; [11]; photochemical reflectance index, PRI; [12]; normalized difference water index, NDWI; [13]; chlorophyll index, CI; [14]; plant senescence reflectance index, PSRI; [15]). Another ever-expanding approach regards the use of multivariate methods, such as partial least squares regression (PLSR), stepwise multiple linear regression (SMLR), principal component regression (PCR) or least absolute shrinkage and selection operator (Lasso) to directly model plant traits as a function of the spectral profile [10]. In contrast to classical regression techniques, PLSR [16] reduces a large number of collinear predictor variables (as in the case with spectral data) into relatively few, uncorrelated latent variables, avoiding the risk of producing unreliable coefficients and error estimates [17], thus becoming the preferred method for chemometric approaches [10,18,19,20]. The model calibration is performed by pairing leaf spectra, collected using a uniform and stable light source and a consistent manner, with independent and reliable measurements. These models are validated on independent samples and can then be applied to other individuals to predict the variable of interest on the basis of their reflectance alone [21]. Several foliar morphological, physiological, and biochemical traits have been successfully estimated from spectra using this approach [18,22,23,24,25,26,27]. Changing perspective, spectra themselves are also a phenotypic expression of the aggregate signals of chemical, morphological, and physiological properties of leaves under specific environmental pressures and management proceedings [28]. As a consequence, spectral characteristics of plants measured across a wide range of wavelengths could potentially provide important information for crop selection and management (i.e., analyses of spectral signatures), more than focusing on individual traits that are often not sufficient to identify and manage crop productivity and quality.

The capability of spectroscopy to monitor the impact of air pollution on vegetation is not a recent discovery, since the first studies date back to the late 70s and early 80s (e.g., [29,30]). Spectroscopy studies about the plant/ecosystem–O_3_ interaction were recently reviewed by Cotrozzi et al. [10]. However, questions remain regarding the capability of the technique to detect and monitor O_3_ stress in plants since (i) most of these studies were performed using limited spectral regions, due to the lack of sensitivity of sensors and portability of instrumentations; (ii) none of these works combined the available approaches to exploit information from spectra (e.g., spectral indices, multivariate-methods to predict standard leaf traits, analyses of spectral signatures); (iii) only chronic O_3_ effects have been studied; and (iv) few species have been examined so far [10].

A number of aromatic plants have been investigated (using standard techniques) for the presence of specialized metabolites that play a key role in plant defense against abiotic and biotic stresses [8,31]. Among them, *Salvia officinalis* L. (sage), an aromatic herb belonging to the Lamiaceae family and largely grown in the Mediterranean region, has been studied because of its important biological properties. In particular, sage contains antioxidant molecules that play an important role against human diseases [32,33]. This is a species with good level of tolerance against salinity [34] and drought [35]. Recent studies have also demonstrated that sage is able to activate biochemical compounds, including phytohormones, signaling molecules, and antioxidants, in order to overcome O_3_-induced oxidative pressure, and for that it can be considered as O_3_ tolerant [36,37].

Here, we test the capability of reflectance spectroscopy to rapidly and non-destructively monitor the responses of asymptomatic sage plants exposed to an acute O_3_ exposure. Specifically, the aims of this work were (i) to develop spectroscopic models for the estimation of a variety of traits related to photosynthesis, the oxidative pressure induced by O_3_, and the antioxidant mechanisms adopted by plants to cope with the pollutant, (ii) to evaluate the potential of spectral phenotyping to accurately detect and predict O_3_ stress, and (iii) to elucidate the variations of both vegetation indices and the abovementioned leaf traits predicted from spectra by PLSR-models, under O_3_ exposure.

## 2. Results

### 2.1. Prediction of Leaf Traits

Several different wavelength ranges (Table S2) and number of components were initially tested to optimize PLSR statistical outputs (i.e., to get highest model goodness-of-fit (*R*^2^) and lowest root mean square error (RMSE) and bias) for the estimations of CO_2_ assimilation rate (A), transpiration (E), stomatal conductance (g_s_), intercellular CO_2_ concentration (C_i_), instantaneous (WUE_i_) and intrinsic (WUE_in_) water use efficiencies, instantaneous carboxylation efficiency (*k*), leaf temperature (T_l_), malondialdehyde (MDA), oxygen radical absorption capacity (ORAC), hydroxyl radical antioxidant capacity (HORAC), oxidized ascorbate (DHA), oxidized:total ascorbate ratio (DHA/ASA_TOT_), reduced glutathione (GSH), total glutathione (GSH_TOT_), chlorophyll *a* (Chl *a*), total chlorophyll (Chl_TOT_), carotenoids (Car), and total phenols (Phen). Final models utilized the following wavelength ranges: 400–1000 nm for A and *k*; 400–2400 nm for E, WUE_i_, WUE_in_, T_l_, HORAC, GSH_TOT_ and Chl_TOT_; 950–2400 nm for g_s_ and C_i_; 400–750 + 1400–2400 for MDA; 1400–2400 nm for ORAC; 1100–1800 nm for DHA and DHA/ASA_TOT_; 400–900 nm for GSH; 400–700 + 1600–1800 nm for Chl *a*; 1100–1400 nm for Car; 1100–1600 nm for Phen (Table 1). 

**Figure 1:**
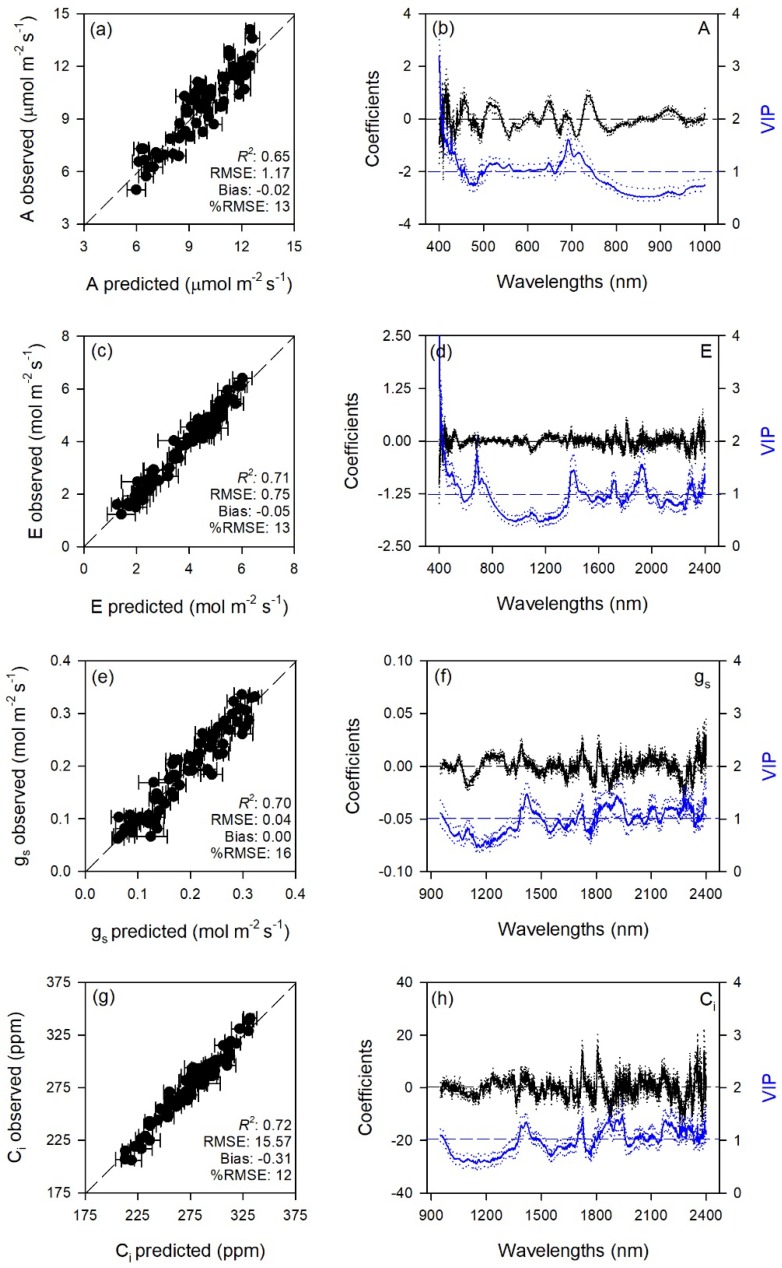
(**a**,**c**,**e**,**g**) Observed versus partial least squares regression (PLSR)-predicted values of CO_2_ assimilation rate (A), transpiration (E), stomatal conductance (g_s_), and intercellular CO_2_ concentration (C_i_) in sage; error bars for predicted values represent the standard deviations generated from 500 simulated models; dashed line is 1:1 relationship; model goodness-of-fit (*R*^2^), root mean square error (RMSE), bias and %RMSE for validation data generated using 80% of the data for calibration and 20% for validation are reported. (**b**,**d**,**f**,**h**) Mean (solid), 5th and 95th percentile (dotted) of standardized coefficients (black), and variable importance for projection values (VIP, blue) by wavelengths for PLSR-models predicting A, E, g_s_, and C_i_ in sage.

**Figure 2:**
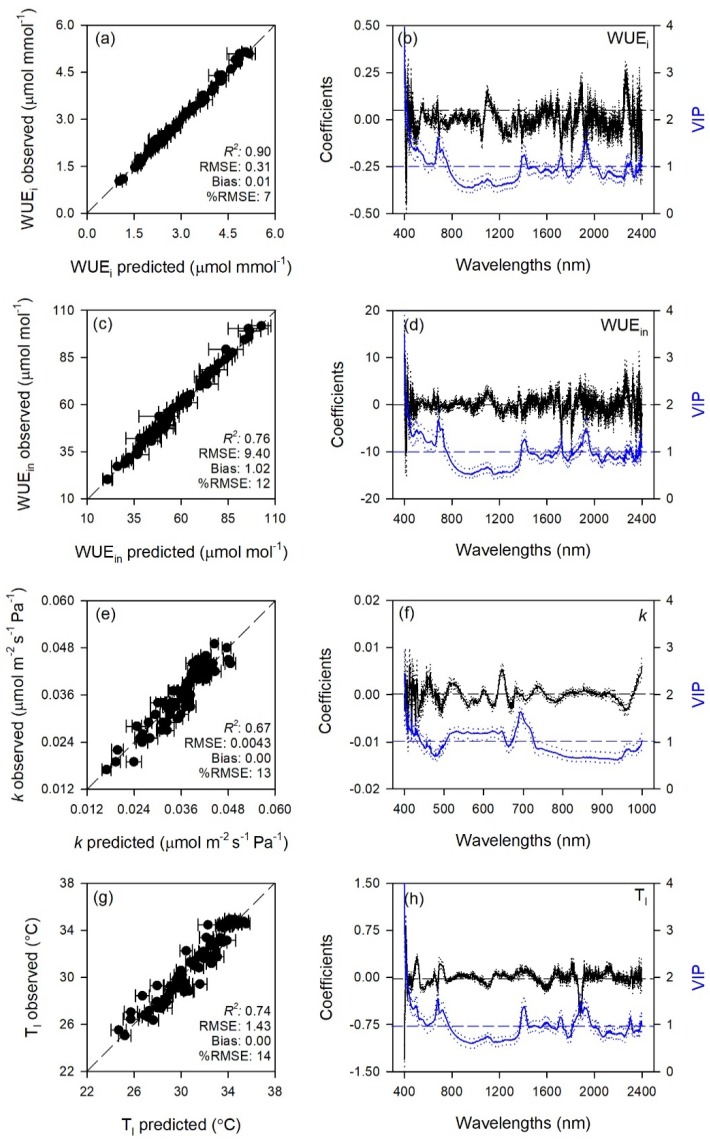
(**a**,**c**,**e**,**g**) Observed versus partial least squares regression (PLSR)-predicted values of intrinsic water use efficiency (WUE_i_), instantaneous water use efficiency (WUE_in_), CO_2_ assimilation rate:intercellular CO_2_ concentration ratio (*k*) and temperature of adaxial leaf surface (T_l_) in sage; error bars for predicted values represent the standard deviations generated from 500 simulated models; dashed line is 1:1 relationship; model goodness-of-fit (*R*^2^), root mean square error (RMSE), bias and %RMSE for validation data generated using 80% of the data for calibration and 20% for validation are reported. (**b**,**d**,**f**,**h**) Mean (solid), 5th and 95th percentile (dotted) of standardized coefficients (black), and variable importance for projection values (VIP, blue) by wavelengths for PLSR-models predicting WUE_i_, WUE_in_, *k*, and T_l_ in sage.

**Figure 3:**
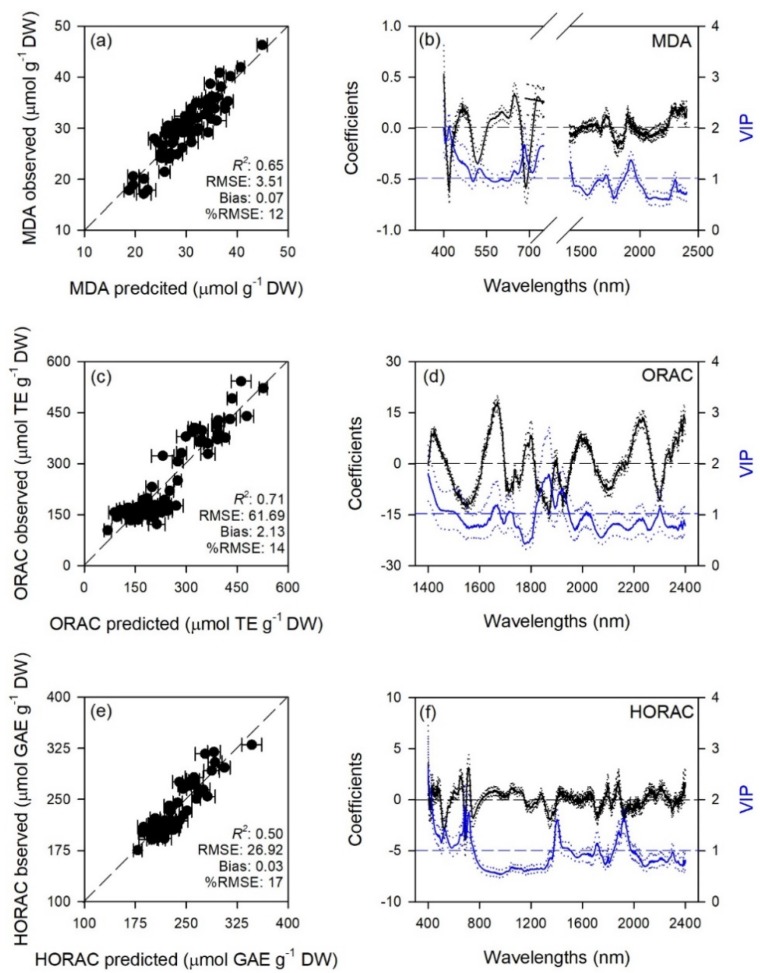
(**a**,**c**,**e**) Observed versus partial least squares regression (PLSR)-predicted values of malondialdehyde (MDA), oxygen radical absorption capacity (ORAC), and hydroxyl radical antioxidant capacity (HORAC) in sage; error bars for predicted values represent the standard deviations generated from 500 simulated models; dashed line is 1:1 relationship; model goodness-of-fit (*R*^2^), root mean square error (RMSE), bias and %RMSE for validation data generated using 80% of the data for calibration and 20% for validation are reported. (**b**,**d**,**f**) Mean (solid), 5th and 95th percentile (dotted) of standardized coefficients (black), and variable importance for projection values (VIP, blue) by wavelengths for PLSR-models predicting MDA, ORAC, and HORAC in sage.

**Figure 4:**
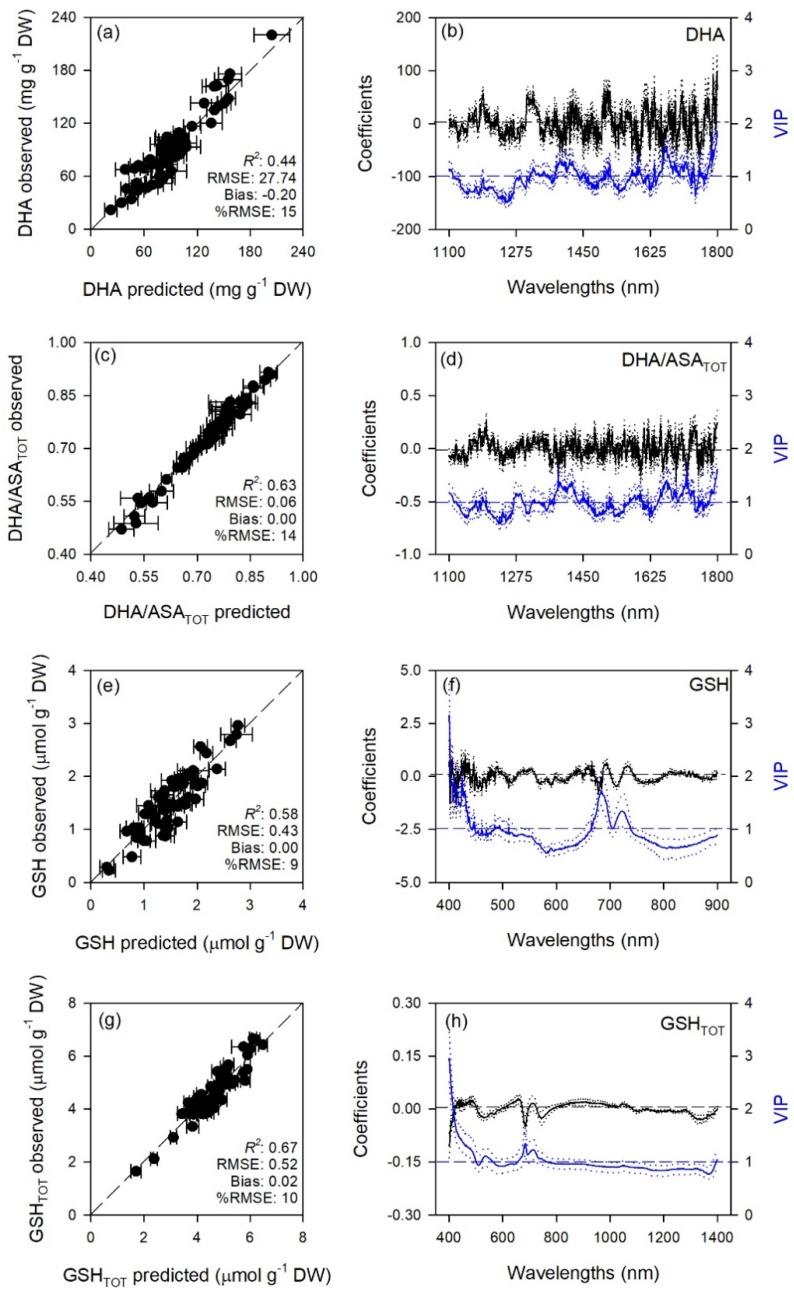
(**a**,**c**,**e**,**g**) Observed versus partial least squares regression (PLSR)-predicted values of oxidized ascorbate (DHA), oxidized:total ascorbate ratio (DHA/ASA_TOT_), reduced glutathione (GSH), and total glutathione (GSH_TOT_) in sage; error bars for predicted values represent the standard deviations generated from 500 simulated models; dashed line is 1:1 relationship; model goodness-of-fit (*R*^2^), root mean square error (RMSE), bias and %RMSE for validation data generated using 80% of the data for calibration and 20% for validation are reported. (**b**,**d**,**f**,**h**) Mean (solid), 5th and 95th percentile (dotted) of standardized coefficients (black), and variable importance for projection values (VIP, blue) by wavelengths for PLSR-models predicting DHA, DHA/ASA_TOT_, GSH, and GSH_TOT_ in sage.

**Figure 5:**
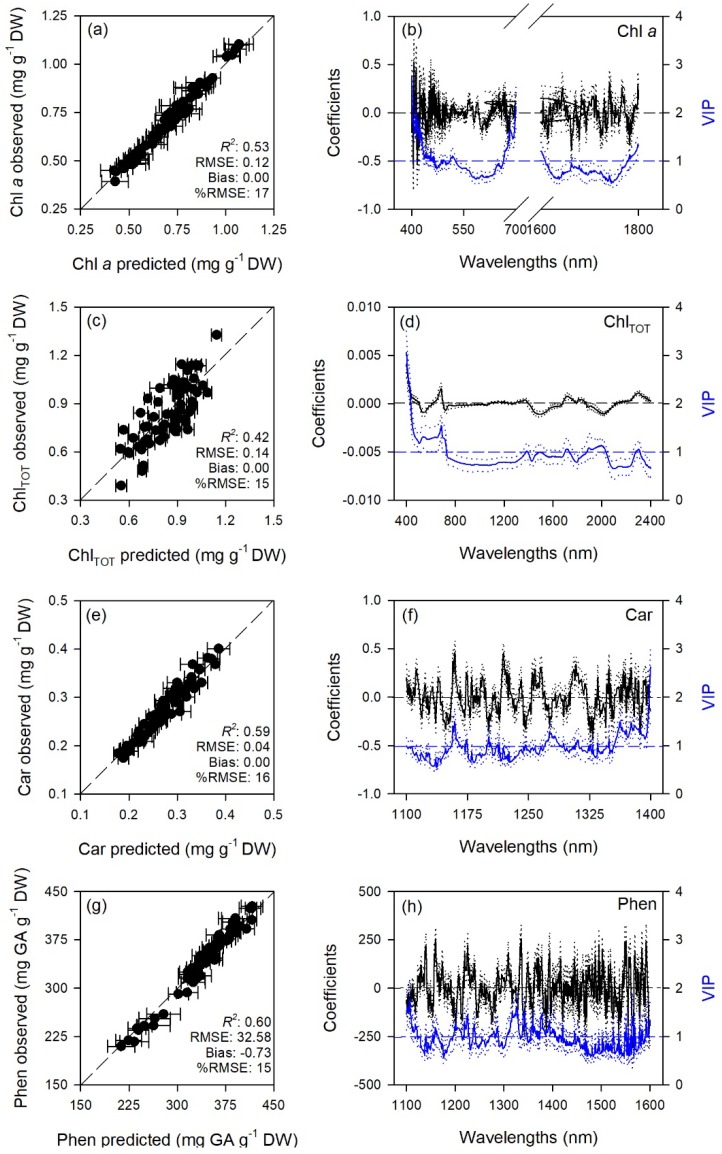
(**a**,**c**,**e**,**g**) Observed versus partial least squares regression (PLSR)-predicted values of chlorophyll *a* (Chl *a*), total chlorophyll (Chl_TOT_), carotenoids (Car), and total phenols (Phen) in sage; error bars for predicted values represent the standard deviations generated from 500 simulated models; dashed line is 1:1 relationship; model goodness-of-fit (*R*^2^), root mean square error (RMSE), bias and %RMSE for validation data generated using 80% of the data for calibration and 20% for validation are reported. (**b**,**d**,**f**,**h**) Mean (solid), 5th and 95th percentile (dotted) of standardized coefficients (black), and variable importance for projection values (VIP, blue) by wavelengths for PLSR-models predicting Chl *a*, Chl_TOT_, Car, and Phen in sage.

Predictive models very accurately characterized gas exchange traits with average model goodness-of-fit (*R*^2^) for validation of 0.65 for A, 0.80 for E, 0.70 for g_s_, 0.72 for C_i_, 0.90 for WUE_i_, 0.76 for WUE_in_, 0.67 for *k*, and 0.80 for T_l_. The RMSEs for validation data were from 1.4 to 3.6 fold higher than RMSEs for calibration data, while larger differences were for WUE_i_ and WUE_in_ (6.2 and 16 fold). Lower prediction performance metrics were found for biochemical traits with average *R*^2^ for validation of 0.65 for MDA, 0.71 for ORAC, 0.50 for HORAC, 0.45 for DHA, 0.63 for DHA/ASA_TOT_, 0.58 for GSH, 0.67 for GSH_TOT_, 0.53 for Chl *a*, 0.42 for Chl_TOT_, 0.59 for Car, 0.61 for Phen. The RMSEs for validation data were from 1.2 to 2.6 fold higher than RMSEs for calibration data, while larger differences were for DHA/ASA_TOT_, Car and Phen (6.0, 4.0, and 4.6 fold). Other performance statistics of PLSR-models are shown in Table 1 and Figure 1, Figure 2, Figure 3, Figure 4 and Figure 5.

Standardized coefficient and variable important to the projection (VIP) statistic profiles highlighted important wavelengths for prediction from 450 to 750 nm for A and *k*, around 700, 1450, and 1900 nm for E, WUE_i_, WUE_in_, and T_l_, and around 1400 and 1900 nm for g_s_ and C_i_ (Figure 1 and Figure 2). For biochemical traits, standardized coefficients and VIP were most pronounced around 700 and 1900 nm for MDA, around 1400 and 1900 nm for ORAC, around 700, 1400, and 1900 nm for HORAC, around 400 and 700 nm for GSH and GSH_TOT_, and around 700 nm for Chl *a* and Chl_TOT_. Given the large number of peaks observed in standardized coefficients and VIP profiles of DHA, DHA/ASA_TOT_, Car and Phen, it was not possible to highlight specific wavelengths more important than others for predictions of these traits (Figure 3, Figure 4 and Figure 5).

### 2.2. Analyses of Spectral Signatures

**Figure 6:**
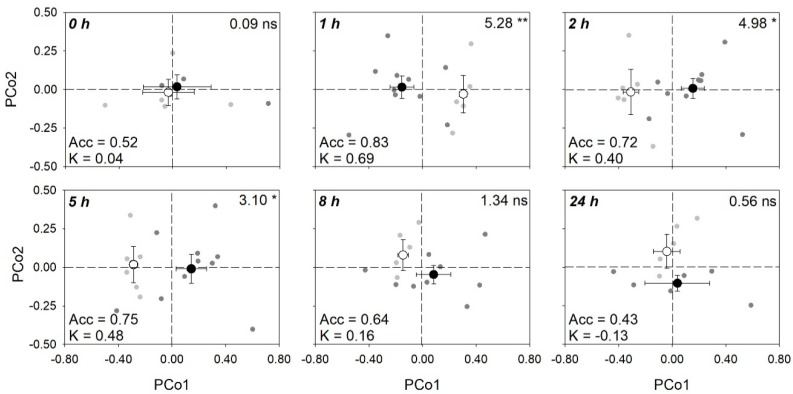
Scores (mean ± standard error) for the first and second principal components from principal coordinates analysis (PCoA) of reflectance data (400–2400 nm) collected from sage leaves, highlighting the capability of spectroscopy to discriminate control (open circle) versus ozonated (200 ppb for 5 h, closed circle) plant groups at 0, 1, 2, 5, 8, and 24 h from the beginning of exposure (gray and dark gray scatter points represent scores for individual control and ozonated samples, respectively). For each time, (i) F- and *p*-values from permutational analysis of variance (PERMANOVA) for the effects of ozone on full-range (400–2400 nm) reflectance profiles of sage leaves are shown in the top-right corners of panels; (ii) average accuracy (Acc) and *kappa* (K) values from partial least squares discriminant analysis (PLS-DA, see Table 3) are shown on the bottom-left corners of panels.

A number of different spectral ranges (Table S2) were examined to optimize the statistical outputs (i.e., to get highest significances for the tested effects on reflectance profiles) of the permutational multivariate analysis of variance (PERMANOVA), but the best results were obtained using the full range (i.e., 400–2400 nm). Final PERMANOVA revealed that only O_3_ affected reflectance profile of sage, whereas no significant effects were found for time and O_3_ × time (Table 2). However, keeping times of analysis separated, PERMANOVA showed significant O_3_ effects only at 1, 2, and 5 h from the beginning of exposure (FBE), as shown by Figure 6 related to the principal coordinates analysis (PCoA). By partial least squares discriminant analysis (PLS-DA), the best classification of control versus O_3_ plant groups from spectra (higher *kappa*) was found with a 80:20 ratio for calibration:validation data using nine components (accuracy: 0.88; 95% confidential interval of accuracy: 0.59–0.98; *kappa*: 0.74 ± 0.17). According to PERMANOVA, keeping times of analysis separated, good classifications between groups were found at 1, 2, and 5 h FBE (average accuracy: 0.83, 0.72, and 0.75; *kappa*: 0.69, 0.40, 0.48, respectively), whereas lower classification outputs were found at 0, 8, and 24 h FBE (average accuracy: 0.52, 0.64 and 0.43; *kappa*: 0.04, 0.16, −0.13, respectively) (Table 3).

### 2.3. Variations of Vegetation Indices and Leaf Traits Derived from Spectra

The effects of O_3_, time, and their interaction on vegetation indices and selected leaf traits derived from spectra are shown in Table 4. Only leaf traits derived by well-performing PLSR-models were selected (validation *R*^2^ > 0.71 and 0.60 for gas exchange and biochemical traits, respectively). The interaction O_3_ × time and time alone were significant on PRI, WUE_i_, WUE_in_, MDA, DHA/ASA_TOT_, and GSH_TOT_. A significant effect of time alone was only found for C_i_ and Phen, while both O_3_ and time alone were significant on ORAC, but not their interaction. Normalized Difference Vegetation Index, CI, and sPSRI did not show any significance. 

**Figure 7:**
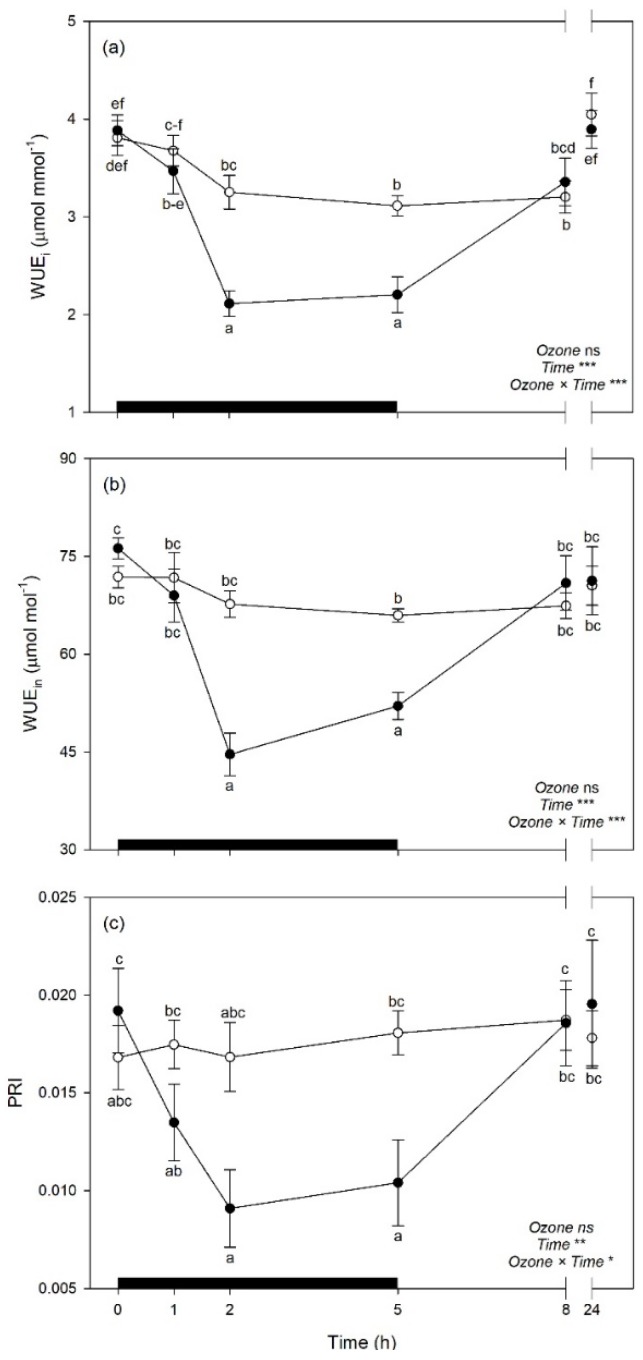
Time course of (**a**) instantaneous water use efficiency (WUE_i_), (**b**) intrinsic water use efficiency (WUE_in_), and (**c**) photochemical reflectance index (PRI) in sage plants exposed to charcoal-filtered air (open circle) or to 200 ppb of ozone for 5 h (closed circle). Data are shown as mean ± standard error. Measurements were carried out at 0, 1, 2, 5, 8, and 24 h from the beginning of exposure. *p*-Values for the effects of ozone (between factor), time (within factor), and their interaction from a one-way repeated measures ANOVA are shown (*** *p* ≤ 0.001; ** *p* ≤ 0.01; * *p* ≤ 0.05; ns: *p* > 0.05). According to Tukey’s post-hoc test, different letters indicate significant differences among means (*p* ≤ 0.05). The thick black bar indicates the time of ozone exposure (i.e., 5 h).

**Figure 8:**
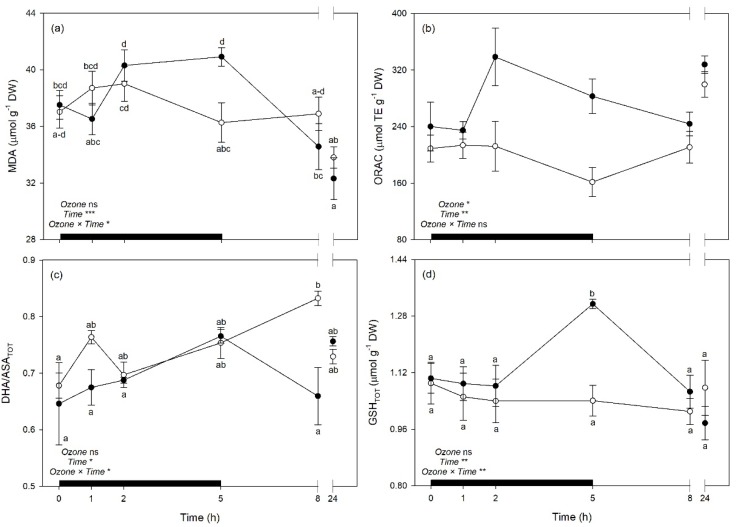
Time course of (**a**) malondialdehyde (MDA), (**b**) oxygen radical absorption capacity (ORAC), (**c**) oxidized:total ascorbate ratio (DHA/ASA_TOT_), and (**d**) total glutathione (GSH_TOT_) in sage plants exposed to charcoal-filtered air (open circle) or to 200 ppb of ozone for 5 h (closed circle). Data are shown as mean ± standard error. Measurements were carried out at 0, 1, 2, 5, 8 and 24 h from the beginning of exposure. *p*-Values for the effects of ozone (between factor), time (within factor), and their interaction from a one-way repeated measures ANOVA are shown (*** *p* ≤ 0.001; ** *p* ≤ 0.01; * *p* ≤ 0.05; ns: *p* > 0.05). According to Tukey’s post-hoc test, different letters indicate significant differences among means (*p* ≤ 0.05). The thick black bar indicates the time of ozone exposure (i.e., 5 h).

Both WUE_i_ and WUE_in_ were reduced by O_3_ exposure at 2 and 5 h FBE (around 33 and 28% in comparison to controls, respectively), while PRI were reduced by the pollutant only at 5 h FBE (−46%); but these traits then recovered at the following times of analysis (Figure 7a–c). Among biochemical traits, MDA showed higher levels in O_3_ plants than controls at the end of the exposure (i.e., 5 h FBE, +13%; Figure 8a), to then come back to control levels. Oxygen Radical Absorption Capacity was generally raised under O_3_ exposure (+28%, averaging times of analyses; Figure 8b). Plants under O_3_ also reduced DHA/ASA_TOT_ ratio at 8 h FBE (−21%, in comparison to controls; Figure 8c), and increased GSH_TOT_ at 5 h FBE (+26%; Figure 8d).

## 3. Discussion

The present study shows an accurate and non-destructive approach by which plant responses to O_3_ can be rapidly detected and monitored using reflectance spectroscopy. Firstly, by combining reflectance measurements, standard physiological and biochemical analyses, and robust statistical modelling, this study demonstrated the potential to concomitantly predict numerous widely used leaf traits related to crop–O_3_ interaction from spectral data.

Photosynthesis is a major plant process that is severely affected by O_3_, primarily through stomatal and mesophyll limitations [1]. Standard measurements of light- and CO_2_-saturated photosynthesis, however, are sometimes logistically challenging, potentially taking several minutes per leaf as the leaf has to acclimate to the cuvette conditions. Spectral approaches present a valid alternative to standard reference measurements and have been used previously to estimate photosynthetic activity in plants both indirectly, via xanthophyll cycling (PRI, [12,38]) and directly, by predicting specific traits from spectral data, mainly RuBP, V_cmax_, and J_max_ (e.g., [39,40,41,42]). Interestingly, we found excellent prediction performance for all the modeled gas-exchange traits (A, E, g_s_, C_i_, WUE_i_, WUE_in_, *k*, and T_l_), with validation *R*^2^ ranging from 0.65 to 0.90 (an external validation of these PLSR-models would be suggested to further test their estimation accuracy, especially for those with larger differences between RMSEs for calibration and validation data). According to the coefficient and VIP profiles, pigment- (450–750 nm) and water-related wavelengths (around 1400 and 1900 nm) were particularly important for these accurate estimations. However, best prediction accuracies (*R*^2^ > 0.75) were found for models using the full range (400–2400; i.e., E, WUE_i_, WUE_in_ and T_l_), followed by those using a narrower range (950–2400 nm; i.e., g_s_ and C_i_), and then by those using an even smaller region (400–1000 nm; i.e., A and *k*). This outcome suggests that the use of wider ranges is a good practice to obtain better predictions of leaf traits from spectra, since (i) using larger ranges means to incorporate more signals of chemical, morphological, and physiological properties of leaves included in the spectra, and (ii) using more prediction variables allows to increase the number of components adopted for modelling without overfit the models. 

However, the use of narrower ranges containing only specific absorption wavelengths for the trait to estimate sometimes leads to better predictions than using the full range, since the incorporation of other spectral regions may reduce the prediction ability of those trait-specific wavelengths [27]. Thus, it is not surprising that the best predictions for A and *k* were found using only the wavelengths from 400 to 1000 nm, with coefficient and VIP profiles highlighting the importance of the region 450–750 nm, since this range includes leaf pigment absorption features [43], as well as the red-edge (700–750 nm; [44]). The importance of these spectral features in the assessment of the photosynthetic processes has been previously reported for several plant species (e.g., [12,39,42,43]), and numerous studies have also shown that the shape of the red-edge is dependent on chlorophyll content (e.g., [45,46]) and stress conditions (e.g., [10,44]). The spectral region best predicting g_s_ and C_i_ was instead in the NIR-SWIR, from 950 to 2400 nm, which is dominated by water content and outside of wavelengths commonly associated with pigments [27]. Thus, the ability to estimate stomatal behavior from spectral data was likely due to the sensitivity of spectra to g_s_-related water regulations. The ability to estimate C_i_ was instead likely ascribable to specific CO_2_-absorption features included in the NIR-SWIR [47], although also this trait could be indirectly associated to water regulations since g_s_ and C_i_ are usually strongly related. These interpretations are supported by the profiles of coefficients and VIP statistics, showing that wavelengths around 1400 and 1900 nm were extremely important for predictions of g_s_ and C_i_. The best ranges found in the present study for predicting gas-exchange traits are not in agreement with our previous findings on maize under drought conditions (best predictions occurred using the 500–900 nm spectral range [48]), suggesting that these outcomes are likely species- and environmental-specific. 

Stomatal closure is considered the first barrier against O_3_ since it limits uptake and preserves plants from oxidative damage, but plants have also evolved enzymatic (e.g., superoxide dismutase, catalase, glutathione peroxidase) and non-enzymatic (e.g., ascorbate, glutathione, phenols, carotenoids) antioxidant systems to cope with considerable amount of reactive oxygen species (ROS) generated by the reaction of O_3_ with the leaf cell apoplast [1,49]. The characterization of these plant–O_3_ interactions by traditional biochemical analyses may be precise, but has several limitations since these analyses are destructive, time consuming and expensive, all aspects that make these methods logistically impractical for monitoring a large number of individual plants. Here, we developed PLSR-models to predict from spectral data a number of widely used leaf traits related to the lipid peroxidation induced by O_3_ (MDA) as well as the antioxidant capacity (ORAC and HORAC) and the main non-enzymatic antioxidants of plants (DHA, DHA/ASA_TOT_, GSH, GSH_TOT_, Car, and Phen), in addition to Chl *a* and Chl_TOT_ having already been largely investigated. However, prediction accuracies for biochemical traits were lower than those reported for gas-exchange parameters (validation *R*^2^ ranging from 0.42 to 0.71), especially considering the higher amount of outliers removed before running final models: only PLSR-models for MDA, ORAC, DHA/ASA_TOT_, GSH_TOT_, and Phen showed acceptable performances (validation *R*^2^ > 0.60), while PLSR-models developed to estimate HORAC, DHA, GSH, and Chl *a* and Chl_TOT_ showed even lower accuracies (validation *R*^2^ < 0.60). Outputs for ORAC and chlorophyll traits, the few parameters already tested by previous studies, were not in accordance with previous reports since Yendrek et al. [42] found low accuracy for a PLSR-model developed to predict ORAC from maize spectra, whereas the ability to estimate chlorophyll contents from spectral data has been demonstrated since long [43]. The inconsistencies between the present study and previous investigations confirm the requirement to develop different models for specific species and environments, as well as to improve the protocols adopted for modeling. Although different ranges were used to best predict the biochemical traits (400–2400 nm for HORAC, GSH_TOT_, and Chl_TOT_; 400–750 + 1400–2400 for MDA; 1400–2400 nm for ORAC; 1100–1800 nm for DHA and DHA/ASA_TOT_; 400–900 nm for GSH; 400–700 + 1600–1800 nm for Chl *a*; 1100–1400 nm for Car; and 1100–1600 nm for Phen), the regions from 400–700 nm and around 1400 and 1900 nm resulted the most important for estimations, as shown by coefficient and VIP profiles. This is in accordance with previous reports about remote sensing of foliar chemistry [10,18,47,50]. Although the approach here proposed may have limitations discriminating fine scale differences in biochemical traits (and we thus encourage caution when interpreting results from a narrow range of values) and an external validation of these PLSR-models (as well as of those for ecophysiological traits) would be suggested to further test their estimation accuracy, the present study also demonstrates the potential to expand prediction capabilities of spectral data for key leaf biochemical features involved in the response of plants to O_3_ pressure.

The prediction of these outcomes, in combination with analyses of spectral signatures, has the potential to provide multiple layers of stress-specific information to growers, including the characterization of the underlying physiological and biochemical responses to O_3_ but also the rapid identification of stress conditions, that can increase the efficiency of management practices. Effectively, the second major achievement of the present study was the demonstration that reflectance profiles of sage plants are sensitive to acute O_3_ exposure. In the absence of visible symptoms, we were able to distinguish with high accuracy sage plants exposed to charcoal-filtered air from those exposed to O_3_ using the full range spectral region (i.e., 400–2400). The utilization of full range spectral data (i.e., a phenotypic expression of the overall physiological, morphological, and biochemical characteristics of leaves under specific environmental conditions; [28]) may prevent loss of information and represent a more powerful approach than direct measurements of leaf traits to monitor crop status. However, the two-way O_3_ × time interactive effect on spectra profiles was not detected. Better outputs might be reached by increasing the experimental/plant replications; this is especially true in field settings, where growing conditions can be highly variable. However, keeping times of analysis separated, we were able to distinguish an O_3_ effect on sage already at 1 h FBE (when none of the traits reported below showed a significant effect), as well as the next two times of analysis, until the end of the O_3_-exposure (i.e., 2 and 5 h FBE). Also, these discriminations resulted in highly accurate results, highlighting the capability of this approach to detect early O_3_ stress, as previously reported for several abiotic and biotic stressors (e.g., [51,52]).

By a phytotoxicologic point of view, the analyses of spectral signatures suggested that O_3_ induced some physiological changes in sage leaves during the exposure (from 1 to 5 h FBE), but plants quickly recovered after the fumigation, confirming the tolerance of this species to the pollutant [36,37]. These responses were confirmed by variations of the investigated vegetation indices and leaf traits derived from spectra (again, only traits from best performing PLSR-models were used), further highlighting the potential of reflectance spectroscopy in monitoring the responses of plants to O_3_. The photosynthetic performance was impaired by the pollutant at 2 and 5 h FBE but recovered to optimal levels after the end of exposure, as confirmed by the trends in WUE_i_, WUE_in_, and PRI (C_i_ was not affected by O_3_). The unchanged values of NDVI, CI, and sPSRI suggest that a senescing process was not induced. Indeed, slightly higher levels of lipid peroxidation (i.e., increased MDA) were only observed at the end of the exposure, and the antioxidant capacity was overall increased by the pollutant (i.e., increased ORAC). The antioxidant response seemed to be finely regulated by the activation/suppression of specific antioxidants at the different times of analysis, both during and after the exposure. A key role in the antioxidant response seemed to be played by the Halliwell-Asada cycle [53], given the O_3_-induced reduction of the DHA/ASA_TOT_ ratio observed at 8 h FBE and the induction of GSH_TOT_ at 5 h FBE; whereas the phenolic metabolism was not likely involved in the response. However, other enzymatic and non-enzymatic antioxidants should be investigated to better highlight how sage cope the O_3_-induced oxidative pressure [49]. Overall, the responses showed by sage to tolerate the acute O_3_ exposure are in accordance with several previous findings (e.g., [37,54,55]).

In conclusion, this study confirms that reflectance spectroscopy can provide a rapid, non-destructive, and relatively inexpensive approach to accurately and concomitantly quantify a number of physiological and biochemical traits in crops associated to the oxidative pressure induced by O_3_ using a single spectral measurement. In addition, it shows that spectral information can successfully identify stress conditions in crops exposed to O_3_ in absence of visible symptoms and using an O_3_-tolerant species, as confirmed by the investigated physiological and biochemical leaf traits. Outcomes and approaches presented in the current study could be of application in a number of scientific fields (e.g., precision agriculture, high-throughput plant phenotyping) with benefits not only for further plant science research, but also for growers to achieve greater crop yield and quality (and incomes), with lower environmental impact. Furthermore, this approach may help to monitor plant function over large geographic regions given its potential capability of being scaled to remote collection from air- or space-borne platforms [10].

## 4. Materials and Methods 

### 4.1. Plant Material and Experimental Design

Experimental activities were performed at the field-station of San Piero a Grado (Pisa, Italy; 43°40′48″ N, 10°20′46″ E, 2 m a.sl.) owned by the Department of Agriculture, Food and Environment of the University of Pisa. On September 2018, thirty-six sage seedlings (4 month-old, 10–15 stems per seedling), grown under field conditions in plastic pots (3.7 L volume) containing a mix of peat and steam-sterilized soil (1:1, *v*/*v*), were selected for uniformity in size (approximately 25 cm tall), equally distributed among four controlled environment fumigation chambers placed inside a walk-in growth chamber, and kept under 22 ± 1 °C of temperature, 85 ± 5% of relative humidity (RH), 500 μmol m^−2^ s^−1^ of photosynthetic active radiation (PAR) provided by incandescent lamps during a 12 h photoperiod, and charcoal-filtered air. The fumigation system was continuously ventilated (two complete air changes per min) with charcoal-filtered air. After two weeks, half of the plants were exposed to 200 ppb of O_3_ (1 ppb = 1.96 µg m^−3^, at 25 °C and 101.325 kPa) for 5 h from 10:00 to 13:00, while the other half of the plants were maintained under charcoal-filtered air (controls, O_3_ concentration < 5 ppb; two chambers per O_3_-treatment). Ozone was generated by a Fisher 500 air-cooled generator (Fisher America Inc., Houston, TX, USA), supplied with pure oxygen, and mixed with the inlet air entering the fumigation chamber; its concentration in the fumigation chambers was continuously monitored with a Serinus 10 analyzer (Ecotech Acoem Group, Milan, Italy). The O_3_ concentration in fumigation chambers used for the high O_3_ treatment was <5 ppb after the end of the exposure. The O_3_ exposure was performed according to Cotrozzi et al. [56] 

Investigations were performed at 0, 1, 2, 5, 8, and 24 h from the beginning of exposure (FBE). Six control plants and six to ten O_3_ plants were dedicated to the analyses of spectral signatures and to the final estimations of leaf traits by PLSR-models developed (see below), thus repeatedly measured, within few minutes and at each time of analysis, only for leaf reflectance and never harvested. The remaining plants were instead dedicated to the development of PLSR-models and, thus, as soon as the previous measures (i.e., those dedicated to the analyses of spectral signatures) were completed, they were consecutively measured for leaf-gas exchange and reflectance and finally harvested (see below). Some of these plants were processed (i.e., gas-exchange-reflectance-harvest) more times (to a maximum of four consecutive times), in order to obtain a total number of 81 gas-exchange measurements and samples for biochemical analyses, coupled with reflectance collections. A summary of the measurement design is reported in Table S1. Gas exchange measurements were performed on one of the second mature and fully expanded leaves (counting from the top of stems), whereas similar and contemporary leaves (three per plant) were harvested, stored at −80 °C, and later freeze-dried for biochemical analyses. Gas exchange analyses made on plants addressed to the development of PLSR-models and measured more times were performed on distinct leaves at each time of analysis. The onset of visible foliar symptoms was constantly monitored.

### 4.2. Collection of Leaf Spectra

Reflectance profiles of leaves were collected using a full range (350–2500 nm) ASD FieldSpec 4 spectroradiometer (Analytical Spectral Devices, Boulder, CO, USA), equipped with a leaf-clip including an internal halogen light source attached to a plant probe. Measurements were made on two areas of the adaxial surface of each leaf, with one measurement per area, and collections were combined to produce an average leaf spectrum. The relative reflectance of each leaf was determined from the measurement of leaf radiance divided by the radiance of a white reference panel included in the leaf-clip, measured every 16 spectral collections.

### 4.3. Foliar Gas-Exchange

The CO_2_ assimilation rate (A), transpiration (E), stomatal conductance (g_s_), intercellular CO_2_ concentration (C_i_), and leaf temperature (T_l_) were determined using a LI-6400 portable photosynthesis system equipped with a 2 × 3 cm chamber and a 6400-02B LED light source (Li-COR Inc., Lincoln, NE, USA), operating at 400 ppm CO_2_ concentration, 25 ± 2 °C of leaf temperature, 45 ± 5 % of RH, 1.8 ± 0.2 kPa of VPD and saturating light conditions (1500 μmol m^−2^ s^−1^ PAR). Instantaneous (WUE_i_) and intrinsic water use efficiencies (WUE_in_) were calculated as A/E and A/g_s_, respectively. Instantaneous carboxylation efficiency (*k*) was calculated as A/C_i_.

### 4.4. Biochemical Analyses

Lipid peroxidation was measured by the TBARS (thiobarbituric acid reactive substances) method, according to Guidi et al. [57]. Briefly, 30 mg of leaf samples were extracted with 750 µL of 80% ethanol, sonicated three times for 10 min and centrifuged at 13,000× *g* for 10 min at 4 °C. Then, 100 µL of each sample supernatant were mixed with 400 µL of 20% trichloroacetic acid (TCA) and 0.5% thiobarbituric acid (TBA). Samples were incubated at 95 °C for 30 min, and centrifuged at 12,000× *g* for 10 min at 4 °C. The supernatant measured for absorbances at 440, 532, and 600 nm, using a Jenway 6505 UV-Vis spectrophotometer (Cole-Parmer, Stone, Staffordshire, UK). The amount of malondialdehyde (MDA) was calculated as 106 × ((A − B)/157,000), where A = ((Abs 532 + TBA) − (Abs 600 + TBA) − (Abs 532-TBA − Abs 600-TBA)) and B = ((Abs 440 + TBA − Abs 600 + TBA) × 0.0571).

The antioxidant properties were tested by measuring the oxygen radical absorption capacity (ORAC) and hydroxyl radical antioxidant capacity (HORAC) according to Ou et al. [58] and Ou et al. [59], respectively. For both analyses, 10 mg of leaf samples, were extracted with 1 mL of 100% methanol, sonicated three times for 10 min, incubated over-night at 4 °C and centrifuged at 13,000× *g* for 15 min at 4 °C. Then, 10 µL of each sample supernatant were mixed with 170 µL of 200 µM fluorescein and incubated at 37 °C for 20 min. The antioxidant scavenging activity was induced by 2,2′-azobis-(2-amidino-propane) dihydrochloride (AAPH) and Co(II) complex, respectively. The antioxidant activity was quantified with excitation at 480 nm and emission at 530 nm, using a Victor3 1420 Multilabel Counter (Perkin Elmer, Waltham, MA, USA). Fluorescence/absorbance was converted to concentration based on an antioxidant standard curve expressed in Trolox (T) equivalents for ORAC and gallic acid equivalents for HORAC.

The total amount of ascorbic acid (ASA_TOT_) and its oxidized form (AsA) were determined according to Kampfenkel et al. [60]. Around 10 mg of leaf samples were extracted with 1 mL of 6% TCA, sonicated three times for 10 min and centrifuged at 12,000× *g* for 10 min at 4 °C. This assay is based on the reduction of ferric ion (Fe^3+^) to ferrous ion (Fe^2+^) with AsA in acid solution, followed by formation of a red chelate between Fe^2+^ and 2,2-dipiridil. Samples were finally read for absorbance at 525 nm with the same spectrophotometer reported above. Absorbances were converted to concentration based on an ascorbic acid standard curve. Concentrations of oxidized ascorbate (DHA) were calculated as ASA_TOT_ − AsA. Oxidized:total ascorbate ratio (DHA/ASA_TOT_) was also calculated.

Total amount of total glutathione (GSH_TOT_) and its oxidized form (GSSG) were determined according to Sgherri and Navari-Izzo [61]. Around 10 mg of leaf samples were extracted with 1 mL of 5% TCA, sonicated three times for 10 min and centrifuged at 12,000× *g* for 10 min at 4 °C. This assay is based on an enzymatic recycling procedure in which glutathione is sequentially oxidized by 5,5′-dithiobis-2-nitrobenzoic acid and reduced by NADPH in the presence of glutathione reductase. The GSSG was determined after removal of GSH from the sample extract by derivatization with 4-vinylpyridine. Absorbances were measured at 412 nm with same spectrophotometer reported above and converted to concentration with a GSH standard curve. Concentrations of the GSH were calculated as GSH_TOT_ − GSSG.

Photosynthetic leaf pigments were measured according to Lichtenthaler [62]. Around 10 mg of leaf material were extracted with 1 mL of 100% acetone, sonicated three times for 10 min and centrifuged at 13,000× *g* for 10 min at 4 °C. The absorbances of sample supernatants were determined at 470, 645, and 662 nm, using the same spectrophotometer reported above. The amounts of chlorophyll *a* (Chl *a*) and *b* (Chl *b*) and Car were calculated as reported by Lichtentaler and Buschmann [63]. Total chlorophyll (Chl_TOT_) was calculated as Chl *a* + Chl *b*.

Total phenols (Phen) were determined according to Ainsworth and Gillepsie [64]. Concisely, 5 mg of leaf samples were extracted with 1.9 mL of 95% methanol, sonicated three times for 10 min, incubated at room temperature for 48 h in the dark and centrifuged at 13,000× *g* for 10 min at 4 °C. Then, 100 µL of sample supernatant were collected and mixed with 200 µL 10% Folin–Ciocalteu reagent and 800 µL of 700 mM Na_2_CO_3_. Samples were incubated at room temperature for 2 h and then measured for absorbance at 765 nm with the same spectrophotometer reported above. Absorbances were converted to concentration with a gallic acid standard curve.

### 4.5. PLSR-Model Calibration and Validation

The PLSR [16] models were generated from untransformed reflectance profiles to predict A, g_s_, C_i_, E, WUE_i_, WUE_in_, *k*, T_l_, MDA, ORAC, HORAC, DHA, DHA/ASA_TOT_, GSH, GSH_TOT_, Chl *a*, Chl_TOT_, Car, and Phen. To avoid potential overfitting, the PLSR models, the numbers of latent variables to use were selected based on reduction of the predicted residual sum of squares (PRESS) statistics [65] using leave-one-out cross-validation. Finally, the selected sets of extracted components were combined into linear models predicting leaf traits based on leaf spectral profiles.

Model performance was evaluated by conducting 500 randomized permutations of the datasets using 80% of the data for calibration and the remaining 20% for validation (internal validation). For each permutation, we calculated statistics to assess model performance when applied to the calibration and the validation data sets: the *R*^2^, the overall error rate (RMSEC, root mean square error; the RMSE for the validation dataset is known as root mean square error for prediction, RMSEP), the percentage of error over the data range (%RMSE), and bias. These randomized analyses generated a distribution of fit statistics allowing for the assessment of model stability as well as uncertainty in model predictions. The strength contribution of PLSR loadings by individual wavelengths was also determined using the variable important to the projection (VIP) statistics. These statistics highlight the importance of individual wavelengths in explaining the variation in both the response and predictor variables, with larger weightings confer greater value to contribution of individual wavelengths to the predictive model [16,66].

Before developing the final modelling, we tested preliminary models to identify poorly predicted outliers. Prediction residuals were used to identify potential outliers, following Couture et al. [18]. Spectral data of outliers were further examined for errors, which included elevated reflectance in the VIS region, misaligned detector splicing producing jumps, or concave spectral shape at the red-edge peak, all a result of the leaf clip is not being fully closed during the reference or target measurements. Standard data of outliers were also examined for extremes in the data distribution. Outliers removed accounted for approximately 10% and 20% of the initial data for gas exchange (less steps and complexity in determinations) and biochemical traits (more steps and complexity in determinations), respectively. The modelling approach and data analyses were performed using the “pls” package in R (www.r-project.org).

Best practices suggest to further external validate the developed PLSR-models (i.e., test their accuracy in prediction on other independent samples not used in model development). This operation was not performed in the current study because of the limitation in sample numerousness. Thus, although outputs for validation are usually in agreement with external validation (e.g., [42,48]), we encourage an external validation before using coefficients from PLSR-models here reported.

### 4.6. Estimation of Leaf Traits by Spectral Indices and PLSR-Models

The following widely-used spectral indices were calculated from spectra: PRI (PRI = (R_531_ − R_570_)/(R_531_ + R_570_); [12]); NDVI (NDVI_570_ = (R_780_ − R_570_)/(R_780_ + R_570_); [11]); CI (CI = (R750 − R705)/(R750 + R705); [14]); PSRI (PSRI = (R_678_ − R_500_)/R_750_; [15]), scaled as sPSRI = (PSRI + 1)/2 to avoid negative values. R_x_ indicates reflectance at x nm wavelength. 

Other leaf traits were calculated by applying the coefficients of the best performing PLSR-models. Both spectral indices and spectra-derived leaf traits were determined from spectra averaged per plant, using plants only dedicated to spectral measurements (as stated above).

### 4.7. Analyses of Spectral Signatures

The influence of O_3_, time and their interactions on the reflectance profiles of sage (averaged per plant) was determined by permutational analysis of variance (PERMANOVA) [67], employing Euclidian measurements of dissimilarity and 10,000 permutations. The same approach was used to determine the influence of O_3_ at each time of analysis (i.e., 0, 1, 2, 5, 8, 24 h FBE). Spectral responses at these times were visualized using Principal Coordinates Analysis (PCoA) on the same spectral data utilized for PERMANOVA, using the “vegan” package in R (www.r-project.org; [68]). Principal Coordinates Analysis uses a distance of uncorrelated variables, or principal coordinates, reducing the dimensionality of the data. 

Partial least squares discriminant analysis (PLS-DA) [69] was additionally used to determine the ability of spectral data to classify experimental groups that showed statistical significance by PERMANOVA. Similar to PERMANOVA, this approach was firstly used on the whole dataset and then on subsets related to separated times of analysis. The analyses were applied 500 times by splitting observations into different groups of calibration (training) and validation (testing) sets. We used the number of correct classifications both in the calibration and the validation sets across the 500 iterations to evaluate the accuracy of the tested model. The calibration:validation data ratio and the number of components called to obtain the models that would give the best fit to the data were determined by iteratively running the PLS-DA models with different calibration:validation data ratios (i.e., 50:50, 70:30, 80:20) and numbers of components and was based on the highest kappa values returned for the validation models. The PLS-DA was performed using the “caret” and “vegan” packages in R (www.r-project.org; [68,70]).

### 4.8. Other Statistical Analyses

Normal distribution of leaf traits derived from spectra (by both spectral indices and PLSR-models) was preliminary analyzed following the Shapiro–Wilk test. The effects of O_3_ (between factor), time (within factor), and their interaction on these leaf traits were tested using a one-way repeated measures analysis of variance (ANOVA). Tukey’s test was used as the post-hoc test. Effects with *p* ≤ 0.05 were considered statistically significant. These univariate statistical analyses were performed in JMP 13.2.0 (SAS Institute Inc. Cary, NC, USA).

## Figures and Tables

**Table 1:** Range, number of latent variables (LV), and model goodness-of-fit (*R*^2^), root mean square error (RMSE), and bias for calibration (cal) and validation (val; i.e., root-mean square error for prediction) data generated using 500 random permutations of the data with 80% used for cal and 20% used for val for PLSR-models predicting leaf traits from sage spectra. Data are shown as mean ± standard deviation. Bias for cal is not shown as it was always <0.001. Trait abbreviations: A, CO_2_ assimilation rate (μmol m^−2^ s^−1^); E, transpiration (mmol m^−2^ s^−1^); g_s_, stomatal conductance (mol m^−2^ s^−1^); C_i_, intercellular CO_2_ concentration (μmol mol^−1^); WUE_i_, instantaneous water use efficiency (μmol mmol^−1^); WUE_in_, intrinsic water use efficiency (μmol mol^−1^); *k*, instantaneous carboxylation efficiency (μmol m^−2^ s^−1^ Pa^−1^); T_l_, temperature of adaxial leaf surface (°C); MDA, malondialdehyde (μmol g^−1^ DW); ORAC, oxygen radical absorption capacity (μmol TE g^−1^ DW); HORAC, hydroxyl radical antioxidant capacity (μmol GAE g^−1^ DW); DHA, oxidized ascorbate (mg g^−1^ DW); DHA/ASA_TOT_, oxidized:total ascorbate ratio; GSH, reduced glutathione (μmol g^−1^ DW); GSH_TOT_, total glutathione (μmol g^−1^ DW); Chl *a*, chlorophyll *a* (mg g^−1^ DW); Chl_TOT_, total chlorophyll (mg g^−1^ DW); Car, carotenoids (mg g^−1^ DW); Phen, total phenols (mg GA g^−1^ DW). An external validation of PLSR-models is suggested to further test their accuracies in estimation of leaf traits.

Trait	Range	LV	Cal		Val		
	(nm)		*R* ^2^	RMSE	*R* ^2^	RMSE	Bias
A	400–1000	11	0.84 ± 0.02	0.81 ± 0.04	0.65 ± 0.13	1.17 ± 0.19	−0.02 ± 0.34
E	400–2400	20	0.97 ± 0.01	0.21 ± 0.02	0.71 ± 0.11	0.75 ± 0.14	−0.05 ± 0.22
g_s_	950–2400	13	0.92 ± 0.01	0.02 ± 0.00	0.70 ± 0.12	0.04 ± 0.01	0.00 ± 0.01
C_i_	950–2400	15	0.96 ± 0.01	5.88 ± 0.51	0.72 ± 0.13	15.57 ± 2.95	−0.31 ± 4.54
WUE_i_	400–2400	22	1.00 ± 0.00	0.05 ± 0.01	0.90 ± 0.06	0.31 ± 0.08	0.01 ± 0.09
WUE_in_	400–2400	25	1.00 ± 0.00	0.59 ± 0.09	0.76 ± 0.14	9.40 ± 2.58	1.02 ± 2.67
*k*	400–1000	13	0.88 ± 0.02	2 × 10^−4^ ± 2 × 10^−4^	0.67 ± 0.14	4 × 10^−4^ ± 7 × 10^−4^	0.00 ± 0.00
T_l_	400–2400	15	0.92 ± 0.01	0.78 ± 0.05	0.74 ± 0.10	1.43 ± 0.25	−0.00 ± 0.43
MDA	400–750 + 1400–2400	10	0.84 ± 0.02	2.33 ± 0.13	0.65 ± 0.15	3.51 ± 0.58	0.07 ± 1.16
ORAC	1400–2400	8	0.85 ± 0.02	45.68 ± 2.51	0.71 ± 0.13	61.69 ± 10.44	2.13 ± 18.18
HORAC	400–2400	12	0.82 ± 0.02	15.36 ± 0.94	0.50 ± 0.19	26.92 ± 4.36	0.03 ± 8.18
DHA	1100–1800	13	0.91 ± 0.02	10.7 ± 1.04	0.44 ± 0.19	27.74 ± 4.02	−0.20 ± 8.31
DHA/ASA_TOT_	1100–1800	16	1.00 ± 0.00	0.01 ± 0.00	0.63 ± 0.15	0.06 ± 0.01	0.00 ± 0.02
GSH	400–900	13	0.90 ± 0.03	0.22 ± 0.01	0.58 ± 0.24	0.43 ± 0.07	0.00 ± 0.13
GSH_TOT_	400–2400	11	0.88 ± 0.02	0.32 ± 0.02	0.67 ± 0.16	0.52 ± 0.09	0.02 ± 0.16
Chl *a*	400–700 + 1600–1800	20	1.00 ± 0.00	0.01 ± 0.00	0.53 ± 0.20	0.12 ± 0.02	0.00 ± 0.04
Chl_TOT_	400–2400	6	0.62 ± 0.04	0.11 ± 0.00	0.42 ± 0.17	0.14 ± 0.02	0.00 ± 0.04
Car	1100–1400	11	0.96 ± 0.01	0.01 ± 0.00	0.59 ± 0.15	0.04 ± 0.01	0.00 ± 0.01
Phen	1100–1600	13	0.98 ± 0.00	7.14 ± 0.90	0.60 ± 0.19	32.58 ± 7.57	−0.73 ± 10.40

**Table 2:** F- and *p*-values of two-way permutational multivariate analysis of variance (PERMANOVA) for the effects of ozone, time, and their interaction on full-range (400–2400 nm) reflectance profiles of sage leaves. *df* represents the degrees of freedom. *** *p* ≤ 0.001, ns: *p* > 0.05.

Effect	*df*	F	*p*
Ozone	1	11.10	***
Time	5	1.05	ns
Ozone × Time	5	0.43	ns

**Table 3:** Latent variables (LV), average accuracy (Acc), 95% confidential interval (CI) of accuracy, and *kappa* (K; mean ± standard deviation) for validation data generated using partial least squares discriminant analysis (PLS-DA), using 80% of the data for calibration and 20% for validation, for the classification of control versus ozonated plant groups from full-range (400–2400 nm) sage spectra, at 0, 1, 2, 5, 8, and 24 h from the beginning of exposure.

Time (h)	LV	Acc	95% CI Acc	K
0	3	0.52	0.04–0.96	0.04 ± 0.64
1	1	0.83	0.20–0.99	0.69 ± 0.35
2	8	0.72	0.15–0.97	0.40 ± 0.57
5	1	0.75	0.16–0.99	0.48 ± 0.44
8	1	0.64	0.10–0.97	0.16 ± 0.41
24	2	0.43	0.02–0.96	−0.13 ± 0.56

**Table 4:** F- and *p*-values of one-way repeated measures analysis of variance (ANOVA) for the effects of ozone (between factor), time (within factor), and their interaction on spectral indices and leaf traits derived from sage spectra (only leaf traits from well performing PLSR-models were used: validation *R*^2^ > 0.70 and 0.60 for gas exchange and biochemical traits, respectively). *df* represents the degrees of freedom. *** *p* ≤ 0.001, ** *p* ≤ 0.01, * *p* ≤ 0.05; ns: *p* > 0.05. See Table 1 for trait abbreviations.

Trait	Ozone (*df*: 1)	Time (*df*: 5)	Ozone × Time (*df*: 5)
PRI	2.46 ns	4.54 **	3.51 *
NDVI	0.15 ns	1.11 ns	0.42 ns
CI	0.30 ns	1.46 ns	0.51 ns
sPSRI	0.36 ns	1.09 ns	0.81 ns
WUE_i_	2.53 ns	76.27 ***	16.96 ***
WUE_in_	1.99 ns	31.66 ***	18.24 ***
C_i_	0.92 ns	68.21 ***	0.54 ns
MDA	0.00 ns	11.15 ***	3.67 *
ORAC	12.54 *	5.29 **	2.23 ns
DHA/ASA_TOT_	3.28 ns	3.51 *	3.61 *
GSH_TOT_	0.95 ns	3.98 **	5.01 **
Phen	0.30 ns	4.59 **	0.35 ns

## Supplementary Materials

The following are available online at https://www.mdpi.com/2223-7747/8/9/346/s1.
