# Supplementary material for: Early Detection of Sage (Salvia officinalis L.) Responses to Ozone Using Reflectance Spectroscopy
Source: Plants (Basel). 2019 Sep 12;8(9):346. doi: 10.3390/plants8090346 (PMC6784234; doi:10.3390/plants8090346)
Supplement: Supplementary file 1 [file plants-08-00346-s001.pdf]

Early Detection of Sage (*Salvia officinalis* L.) Responses to Ozone Using Reflectance Spectroscopy

Table S1. Summary of measurement design showing plants measured (*) at 0, 1, 2, 5, 8, and 24 h from the beginning of the exposure for analyses of spectral signatures (SS) and for PLSR-modelling (PLSR). Treatments (Treatm; control (C) versus ozone (O_3)) and numbers of exposure chambers are also specified.

**Plant**	**Treatm**	**Chamb**	**0 h**		**1 h**		**2 h**		**5 h**		**8 h**		**24 h**	
			SS	*PLSR*	*SS*	*PLSR*	*SS*	*PLSR*	*SS*	*PLSR*	*SS*	*PLSR*	*SS*	*PLSR*
1	C	1	*		*		*		*		*		*	*
2	C	1		*		*		*		*				
3	C	1				*		*		*		*		
4	C	1	*		*		*		*		*		*	*
5	C	1								*		*		
6	C	1						*		*		*		
7	C	1										*		*
8	C	1	*		*		*		*		*		*	*
9	C	2						*		*		*		
10	C	2	*		*		*		*		*		*	*
11	C	2		*		*		*		*				
12	C	2				*		*		*		*		
13	C	2								*		*		
14	C	2		*		*		*		*				
15	C	2	*		*		*		*		*		*	*
16	C	2										*		*
17	C	2		*		*		*		*				
18	C	2	*		*		*			*		*		
19	O_3	3						*		*		*		
20	O_3	3	*		*		*		*		*		*	
21	O_3	3			*		*		*	*		*		
22	O_3	3				*		*		*		*		
23	O_3	3	*		*		*		*		*		*	*
24	O_3	3			*		*		*		*	*		*
25	O_3	3				*		*		*		*		
26	O_3	3	*		*		*		*		*		*	*
27	O_3	3								*		*		
28	O_3	4						*		*		*		
29	O_3	4	*		*		*		*		*		*	*
30	O_3	4			*		*		*		*	*		*
31	O_3	4				*		*		*		*		
32	O_3	4	*		*		*		*		*		*	*
33	O_3	4			*		*		*		*	*		*
34	O_3	4						*		*		*		
35	O_3	4	*		*		*		*		*		*	*
36	O_3	4								*		*		
	Tot C plants		6	4	6	6	6	8	6	10	6	8	6	7
	Tot O_3 plants		6	0	10	3	10	6	10	9	9	12	6	8

Table S2. Tested ranges of (i) preliminary PLSR-models used the estimation of leaf traits by spectral data, and (ii) preliminary PERMANOVA for the effects of ozone, time, and their interaction on reflectance profiles of sage leaves. Final ranges are in bold. Trait abbreviations: A, CO_2 assimilation rate; E, transpiration; g_s, stomatal conductance; C_i, intercellular CO_2 concentration; WUE_i, instantaneous water use efficiency; WUE_{in}, intrinsic water use efficiency; *k*, instantaneous carboxylation efficiency; T_l, temperature of adaxial leaf surface; MDA, malondialdehyde; ORAC, oxygen radical absorption capacity; HORAC, hydroxyl radical antioxidant capacity; DHA, oxidized ascorbate; DHA/ASA_{TOT}, oxidized:total ascorbate ratio; GSH, reduced glutathione; GSH_{TOT}, total glutathione; Chl *a*, chlorophyll *a*; Chl_{TOT}, total chlorophyll; Car, carotenoids; Phen, total phenols.

Trait	Tested Ranges (nm)
A	400–2400, 950–2400, 1400–2400, 400–900, 600–900, 500–1100, 400–1200, 400–1100, **400–1000**
E	**400–2400**, 400–1200, 950–2400, 1100–2400, 1400–2400, 600–900, 500–1100
g_s	400–2400, 400–1200, 1400–2400, **950–2400**
C_i	400–2400, 400–1200, 1400–2400, **950–2400**
WUE_i	**400–2400**, 950–2400, 1400–2400, 400–1200
WUE_{in}	**400–2400**, 400–1200, 500–1100, 600–900, 900–1200
k	400–2400, 950–2400, 1400–2400, 400–1200, 600–900, 500–1100, 400–1100, **400–1000**
T_l	**400–2400**, 950–2400, 1400–2400, 400–900, 600–900
MDA	400–2400, 950–2400, 1100–2400, 1400–2400, 500–1100, 400–1200, 400–750 + 1100–2400, **400–750 + 1400–2400**
ORAC	400–2400, 950–2400, 1100–2400, 950–1800, 1200–1800, 950–1600, 1400–1800, 1800–2250, **1400–2400**
HORAC	400–2400, 950–2400, 1100–2400, 950–1800, 1200–1800, 950–1600, 1400–1800, 1800–2250, **1400–2400**
DHA	400–2400, 950–2400, 1100–2400, 500–1100, 600–900, 950–2250, 950–1800, 1100–1400 + 1600–1800, 1100–1400 + 1800–2250, **1100–1800**
DHA/ASA_{TOT}	400–2400, 500–1100, 600–900, 950–2400, 1400–2400, 1100–2400, **1100–1800**
GSH	400–2400, 950–2400, 1400–2400, 400–1200, 500–1100, 600–900, 400–750, **400–900**
GSH_{TOT}	**400–2400**, 950–2400, 1400–2400, 400–1200, 400–900, 600–900, 500–1100, 400–1800, 900–1200, 1100–2400, 400–700 + 950–2400, 400–700 + 950–1400, 400–1100 + 1800–2400, 400–750 + 1400–2400
Chl *a*	400–2400, 400–1800, 400–1200, 400–900, 600–900, 500–1100, 400–800, 400–700, 400–700 + 900–1200, 400–700 + 1400–1800, **400–700 + 1600–1800**
Chl_{TOT}	**400–2400**, 400–1800, 400–1200, 500–1100, 400–1000, 400–900, 400–800, 600–900, 950–2400, 400–700 + 1100–1800, 400–700 + 950–1800, 400–700 + 1100–2400, 400–700 + 1000–2400, 400–700 + 900–2400, 400–700 + 1400–2400, 400–900 + 1600–2400, 400–900 + 1800–2400, 400–900 + 1200–2400, 400–700 + 1000–2400
Car	400–2400, 400–1200, 500–1100, 600–900, 400–900, 400–700, 400–700 + 1100–1400, 400–600 + 700–800, 950–2400, 950–2250, 950–1400, 1100–1600, 1100–1500, 1200–1800, 1200–1600, 1200–1400, 1000–1400, 1000–1300, **1100–1400**
Phen	400–2400, 950–2400, 1100–2400, 1200–2400, 1400–2400, 1600–2400, 1800–2400, 950–1800, 950–1600, 1100–1400, 400–750 + 1400–2400, 1200–1600, 1000–1600, 1100–1500, **1100–1600**
PERMANOVA	**400–2400**, 400–750, 950–2400, 1400–2400, 2000–2400, 400–1200, 400–1800, 950–1600, 1200–1800, 1400–1800, 400–1400 + 1800–2250, 400–750 + 1200–1600, 400–750 + 950–1200, 400–750 + 1000–2400
